# Association between increased serum alkaline phosphatase and the coronary slow flow phenomenon

**DOI:** 10.1186/s12872-018-0873-6

**Published:** 2018-07-04

**Authors:** Yong Wang, Mou-jie Liu, Hui-min Yang, Chun-yan Ma, Peng-yu Jia, Da-lin Jia, Ai-jie Hou

**Affiliations:** 1 Department of Cardiology, The People’s Hospital of China Medical University, The People’s Hospital of Liaoning Province, No. 33, Wenyi road, Shenhe District, Shenyang City, Liaoning Province China; 2 Department of Cardiology, The First Affiliated Hospital of China Medical University, No. 155, Nanjing road, Heping District, Shenyang City, Liaoning Province China; 3 Department of Cardiovascular Ultrasound, The First Affiliated Hospital of China Medical University, No. 155, Nanjing road, Heping District, Shenyang City, Liaoning Province China

**Keywords:** Serum alkaline phosphatase, TIMI flame count, Coronary slow flow phenomenon, Coronary angiography

## Abstract

**Background:**

Despite marked advances in our understanding of the pathophysiology of the coronary slow flow phenomenon (CSFP), the exact mechanism remains unclear. Previous studies have suggested that CSFP might be associated with generalized atherosclerosis, endothelial dysfunction, and low-grade chronic inflammation. High serum alkaline phosphatase (ALP) levels are associated with vascular calcification, atherosclerotic disease, and an increased risk of cardiovascular events. However, the relationship between ALP and CSFP is unclear.

**Methods:**

We investigated 64 patients with angiographically proven CSFP and 50 with normal coronary flow. Serum ALP levels were measured in all studied individuals.

**Results:**

Serum ALP levels in patients with CSFP were significantly higher than those in the control group (70.5 ± 17.1 vs. 61.9 ± 16.1 U/L, *P* = 0.007). A positive association was observed (*r* = 0.42, *P* = 0.032) between serum ALP levels and the mean thrombolysis in myocardial infarction frame count (mTFC). Regression analysis showed a high serum ALP level was the only independent predictor of the mTFC (β = 0.309, *P* < 0.001). Moreover, our study showed that a serum ALP level > 67.5 U/L was a predictor of CSFP (sensitivity = 83.3%, specificity = 84.1%).

**Conclusions:**

Patients with CSFP show high serum ALP levels, which may be associated with the pathogenesis of CSFP. A high serum ALP level is a predictor of CSFP. Future studies are needed to clarify the role of ALP in patients with CSFP.

## Background

The coronary slow flow phenomenon (CSFP) is characterized by normal or near-normal epicardial coronary arteries (stenosis < 40%) but delayed distal vessel opacification using a contrast agent during diagnostic coronary angiography [1]. This angiographic entity was first discovered by Tambe et al. in 1972 [2]. CSFP is not merely an angiographic discovery because a growing body of evidence has demonstrated that CSFP may lead to angina pectoris, ventricular tachycardia/fibrillation, acute myocardial infarction, and even sudden cardiac death [3–6]. Microvascular [7] and endothelial dysfunction [8], which have been reported in several studies, along with chronic inflammation [8–11] and diffuse atherosclerosis are [12] considered underlying etiologies associated with CSFP; however the exact etiology of CSFP remains unclear.

Alkaline phosphatase (ALP) is a membrane-bound glycoprotein (metalloenzyme) showing widespread expression in human tissues, with the highest activity observed in the liver, bone, and the kidneys [13]. Similar to the role of C-reactive protein (CRP), ALP has been reported as a novel risk marker (inflammatory mediator) for cardiovascular disease [14] with a proven association between elevated serum ALP levels and coronary atherosclerosis [13–15]. Because the pathogenesis of CSFP and the role of ALP is similar, we investigated the relationship between the 2 and attempted to suggest new insights into the development of CSFP.

## Methods

### Patients

Our study included patients who visited the First Affiliated Hospital of China Medical University between December 2014 and March 2016. Angiographic records of 3345 patients who underwent coronary angiography were evaluated prospectively by 2 experienced interventional cardiologists. All patients complained of chest pain but coronary angiography showed normal or near-normal epicardial coronary arteries (stenosis < 40%) in all. The cut-off values of the thrombolysis in myocardial infarction frame count (TFC) for the normal filling of epicardial coronary arteries were 36.2 ± 2.6 for the left anterior descending artery (LAD) (corrected cut-off value for LAD was 21.1 ± 1.5), 22.1 ± 4.1 for the left circumflex artery (LCX), and 20.4 ± 3 for the right coronary artery (RCA) [16]. Patients with values above this criterion were defined as showing CSFP, and individuals showing values below this criterion were defined as the normal control group. Finally, 64 patients (29 men, 35 women, mean age 58.6 ± 7.9 years) with CSFP and 50 controls (24 men, 26 women, mean age 57.4 ± 8.4 years) were included in this study. This study was approved by the Ethics Committee of the hospital, and informed consent was obtained from all individuals who participated in the study.

Exclusion criteria were: patients with previous coronary heart disease (recent/prior myocardial infarction, or acute coronary syndrome), heart failure, valvular heart disease, cardiomyopathy, non-sinus rhythm, severe systemic disease, and/or alcohol consumption.

### Blood sampling

Blood samples were obtained from a forearm vein after a 12-h fast before performing coronary angiography. Routine blood tests were performed to assess the complete blood count, blood glucose, uric acid (UA), ALP, and gamma-glutamyl transferase levels, a lipid profile, and renal function. Blood tests were performed as a routine procedure in the Laboratory Department of our hospital. Serum ALP was measured using a Hitachi model 737 multichannel analyzer (Roche Diagnostics, Indianapolis, IN). The adult reference range was 39–117 U/L).

### Coronary angiography

A standard Judkins technique was used in all the studied individuals. At least 4 and 2 views, respectively, were obtained for the left and right coronary arteries. Iohexol (contrast agent) was injected at a rate of 3–4 mL/s for the left coronary artery and 2–3 mL/s for the RCA. Coronary angiography was performed at a rate of 30 frames/s. Angiograms were assessed by 2 experienced cardiologists who were blinded to the clinical details of the subjects. The TFC was used to quantitatively evaluate coronary blood flow. The initial frame was defined by a column of contrast extending across > 70% of the arterial lumen in an antegrade fashion [16]. The final frame was designated when the leading edge of the contrast column appeared at the distal end. The distal end was defined as the distal bifurcation for the LAD, the distal bifurcation of the segment with the longest total distance for the LCX, and the first branch of the posterolateral artery for the RCA. The corrected TFC for the LAD was derived from the original value after dividing it by 1.7 [17]. The corrected cut-off values (owing to the length for normal visualization of coronary arteries) were 36.2 ± 2.6 for the LAD, 22.2 ± 4.1 for the LCX, and 20.4 ± 3 frames for the RCA [16]. Values > these thresholds indicated CSFP.

### Statistical analysis

Continuous variables were expressed as means±standard deviation, and categorical variables were expressed as percentages. Intergroup differences were tested using the Student t- and theχ^2^ tests for continuous and categorical data, respectively. The Spearman correlation coefficient was used to assess the association between variables. Regression analysis was used to detect the predictors of CSFP. A univariate regression model was used separately for each of the following covariates: sex, body mass index, hypertension, smoking, dyslipidemia, mean platelet volume (MPV), cystatin C, UA, and ALP. Covariates that showed significant associations with CSFP using the univariate model were subjected to multivariate logistic regression analysis. Confounders showing statistical significance at the *P* < 0.05 level were subjected to regression analysis. A *P* value < 0.05 was considered statistically significant.

## Results

This study included 114 patients. Demographic characteristics of patients are listed in Table 1. No statistically significant intergroup differences were observed with regard to age, sex, body mass index, current smoking habits, blood pressure, dyslipidemia, and diabetes mellitus. Medication use did not differ between patients with CSFP and normal controls.

**Table 1:** Baseline clinical characteristics of the two groups

	CSFP group (*n* = 64)	Control group (*n* = 50)	*P* value
Age (years)	58.6 ± 7.9	57.4 ± 8.4	0.25
Males, n(%)	29(45.3)	24(48.0)	0.85
BMI, (kg/m^2^)	24.7 ± 3.2	25.2 ± 5.3	0.59
Current smoker, n(%)	9(14.1)	6(12.0)	0.79
Dyslipidemia, n (%)	10(15.6)	6(12.0)	0.79
Diabetes mellitus, n(%)	9(14.1)	5(10.0)	0.58
systolic blood pressure, (mmHg)	123.7 ± 15.0	125.4 ± 13.2	0.62
Diastolic bloodpressure, (mmHg)	77.7 ± 9.4	81.7 ± 10.9	0.11
Medications			
Aspirin, n(%)	40(62.5)	32(64.0)	0.29
Statin, n(%)	38(59.4)	32(64.0)	0.70
ACEI/ARB, n(%)	8(12.5)	9(18.0)	0.44
CCB, n(%)	6(9.4)	7(14.0)	0.56
Nitrates, n(%)	36(6.3)	30(60.0)	0.71

Values are mean ± standard deviation or numbers with percentages in parentheses

*ACEI* angiotensin-converting enzyme inhibitor, *ARB* angiotensin II receptor blocker, *CCB* calcium antagonist

Angiographic characteristics of patients with CSFP are shown in Table 2. In this study, a total of 22 (34.4%) patients showed CSFP in the LAD, 33 (51.6%) in the LCX, and 42 (65.6%) in the RCA. Notably, 18 patients showed CSFP in 3 main coronary arteries, 23 in 2, and 23 in 1.

**Table 2:** Angiographical characteristics of the patients with CSFP

TFC in Each Artery			
TFC(LAD)	44.7 ± 13.3		
TFC(LCX)	30.3 ± 10.7		
TFC(RCA)	34.5 ± 15.4		
mean TFC	36.5 ± 11.2		
Vessel Involvement			
One (%)	23(35.9)		
Two (%)	23(35.9)		
Three (%)	18(28.1)		
Coronary Artery Involvement			
LAD(n, %)	22(34.4)		
LCX(n, %)	33(51.6)		
RCA(n, %)	42(65.6)		

Values are mean ± standard deviation or numbers with percentages in parentheses

*TFC* TIMI frame count

**Fig. 1:**
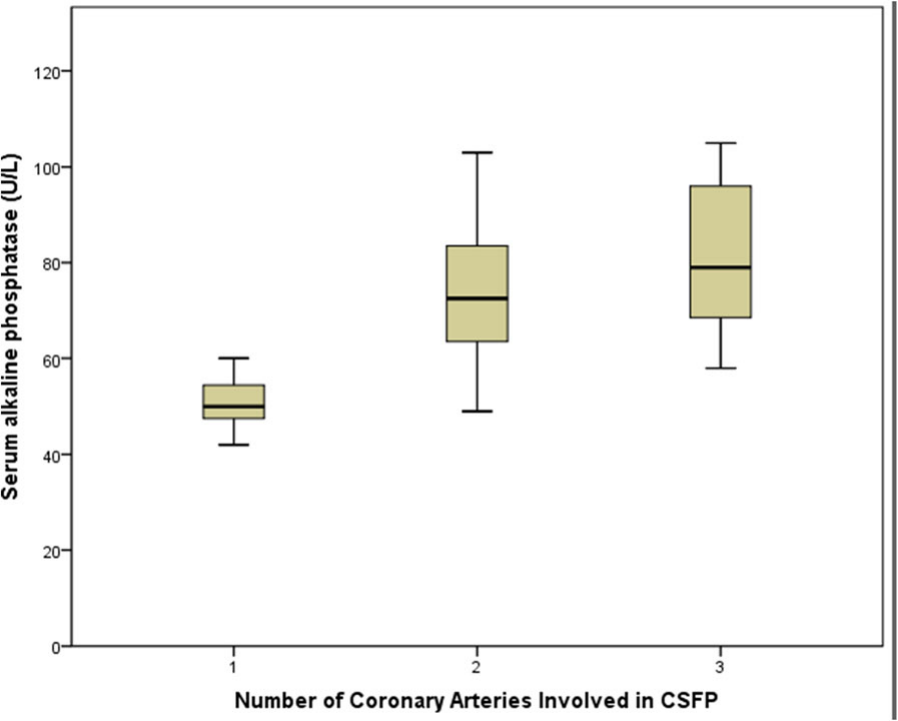
Correlation between the number of coronary arteries involved in CSFP and serum ALP level

**Fig. 2:**
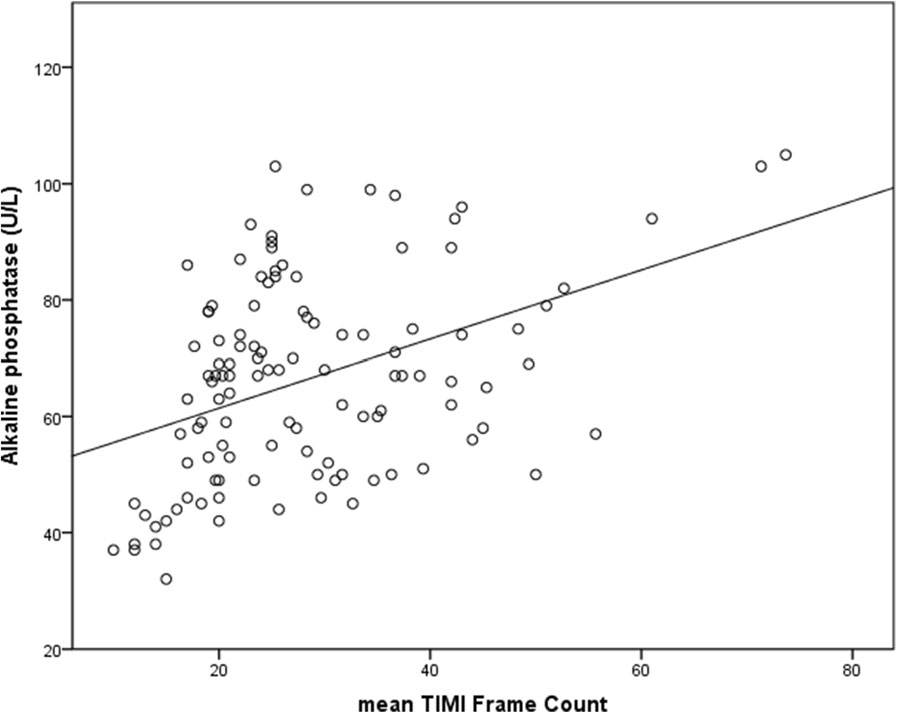
Correlation between mean TFC and serum ALP level

Laboratory parameters including fasting levels of blood glucose, low-density lipoprotein, creatinine, UA, and white blood cells were comparable between groups (Table 2). The serum ALP level was significantly higher in the CSFP than in the control group (70.5 ± 17.1 vs. 61.9 ± 16.1 U/L *P* = 0.007, Table 3) and its level increased with an increase in the number of vessels involved (Fig. 1). A statistically significant positive association was observed between the mean thrombolysis in myocardial infarction frame count (mTFC) and the serum ALP level (*r* = 0.42, *P* < 0.001, Fig. 2), UA (*r* = 0.207 *P* = 0.033, Table 4), and the MPV (*r* = 0.213 *P* = 0.031, Table 4). Multiple linear regression analysis showed that the serum ALP level was an independent predictor of CSFP (Table 4).

**Table 3:** Baseline laboratory characteristics of the two groups (mean ± SD)

	CSFP group (*n* = 64)	Control group (*n* = 50)	*P* value
WBC(10^9^/L)	6.8 ± 1.6	6.2 ± 1.5	0.56
RDW(%)	13.2 ± 0.8	12.7 ± 0.6	0.37
MPV(fl)	10.5 ± 1.1	10.3 ± 0.8	**0.04**
PDW(%)	11.2 ± 1.1	10.7 ± 0.8	0.45
HDL-C(mmol/l)(mmol/l)	1.0 ± 0.4	1.1 ± 0.6	0.46
LDL-C(mmol/l)	2.5 ± 0.8	2.8 ± 0.7	0.20
TG(mmol/l)	1.4 ± 1.0	1.3 ± 0.9	0.67
TC(mmol/l)	4.3 ± 0.7	4.5 ± 0.9	0.22
FG (mmol/L)	5.4 ± 0.8	5.7 ± 0.7	0.18
Creatinine(mg/dl)	57.7 ± 11.2	55.5 ± 13.9	0.29
Cys-C(mg/l)	1.0 ± 0.3	0.9 ± 0.1	0.31
UA(umol/l)	309.5 ± 72.5	301.2 ± 62.5	0.64
GGT(U/l)	33.0 ± 28.5	35.0 ± 32.2	0.82
ALP(U/l)	70.5 ± 17.1	61.9 ± 16.1	**0.007**

*RDW* red cell distribution width, *MPV* mean platelet volume, *PDW* platelet distribution width, *HDL* high-density lipoprotein, *LDL* low-density lipoprotein, *TG* Triglyceride, *TC* total cholesterol, *FG* fasting glucose, *Cys-C* Cystatin C, *UA* uric acid, *GGT* gamma glutamyl transpeptidase, *ALP* alkaline phosphatase

Bold values are statistically significant (*p* < 0.05)

**Table 4:** Relationship between the mean TIMI frame count and laboratory parameters

Variable	Pearson analysis		Regression analysis	
	r	*P*	β	*P*
Sex	0.214	0.176		
Body Mass Index	0.203	0.143		
Smoking	0.043	0.892		
Hypertension	0.218	0.324		
Dyslipidemia	0.194	0.467		
Diabetes mellitus	0.172	0.526		
MPV	0.213	**0.031**	0.092	0.123
Cys-C	0.303	0.042		
UA	0.207	0.033		
ALP	0.420	**< 0.001**	0.278	**0.032**

*MPV* mean platelet volume, *Cys-C* Cystatin C, *UA* uric acid, *ALP* alkaline phosphatase

Bold values are statistically significant (*p* < 0.05)

**Fig. 3:**
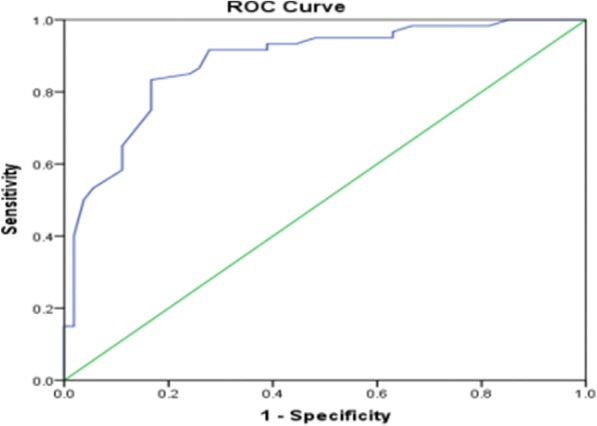
The receiver-operating characteristic(ROC) curve for the discrimination of patients with CSFP at least in one of the coronary arteries from patients with the normar controls

Receiver operating characteristic curve analysis showed that the serum ALP level demonstrated a high diagnostic value in differentiating patients with CSFP from normal controls (area under the curve = 0.881, Fig. 3). The receiver operating characteristic curve showed that a serum ALP level of 67.5 U/L was a predictor of CSFP with a sensitivity of 83.3% and a specificity of 84.1%.

## Discussion

This study showed that the serum ALP level was significantly elevated in patients with CSFP and moderately associated with the mTFC. The serum ALP level increased with an increase in the number of vessels involved. An elevated serum ALP level was an independent predictor of CSFP. To our knowledge, this is the first study in the literature to report a link between serum ALP levels and the pathogenesis of CSFP.

The pathophysiology of CSFP remains unclear. Unlike cardiac X syndrome, CSFP is more common in male smokers, patients with dyslipidemia, metabolic syndrome, and obesity [17]. Although the pathogenesis of CSFP remains unclear, a few underlying mechanisms have been proposed including coronary microvascular disease [2, 18], diffuse atherosclerosis [12], endothelial dysfunction [8], chronic inflammation [8–10], and the role of oxidative stress [8, 19]. Other routine clinical biochemical parameters such as the plateletcrit and red cell distribution width [20], elevated serum lipids [17], cystatin C [21], and UA levels [22] have been proved to be associated with CSFP. Similarly, a higher MPV was observed in our study in patients with CSFP. The heterogeneity in hematological indices suggests that the pathogenesis of CSFP is complex and several factors (medications, the patient’s dietary status, and/or racial differences) may be involved. Further research is warranted to gain a better understanding of the pathogenesis and risks associated with CSFP.

A growing body of evidence suggests that diffuse atherosclerosis may be involved in the pathogenesis of CSFP. Using intravascular ultrasonography, Pekdemir demonstrated that patients with CSFP showed a decreased fractional flow reserve, which was attributable to increased resistance in the epicardial coronary arteries secondary to diffuse atherosclerotic disease [23]. Another study using intravascular ultrasonography [12] reported that patients with CSFP showed diffuse intima-media thickening (IMT) in their coronary arteries; however, they do not cause luminal morphology changes observed by coronary angiography in patients with CSFP. Another study indicated that patients with CSFP showed increased IMT, suggesting the role of atherosclerosis in this phenomenon [24]. Additionally, epidemiological research has demonstrated that increased serum ALP levels are associated with atherosclerosis in coronary, peripheral, and cerebral arteries [13, 15, 25]. An elevated serum ALP level may promote vascular calcification over the pyrophosphate pathway [26, 27], which may damage vascular integrity and promote atherosclerosis, thereby causing widespread involvement of coronary vessels. Vascular calcification is a basic pathway involved in the initiation and progression of atherosclerosis, which arises in vascular sclerosis, aging, and significant cardiovascular events [28]. Our study showed that a significantly elevated serum ALP level was positively associated with the mTFC in patients with CSFP and that the serum ALP level increased with an increase in the number of vessels involved. These findings suggest that atherosclerosis secondary to elevated serum ALP levels may contribute to the pathophysiology of CSFP. Thus, serum ALP may be a useful biomarker to predict the occurrence of CSFP. However, an increased serum ALP level may occur as a pathological product of CSFP. Further investigations are needed to confirm these results.

Serum ALP levels can be influenced by a variety of clinical factors such as age, medication use, liver function, smoking habits, physical inactivity, alcohol consumption, and metabolic syndrome [29]. In this study, these factors were comparable between the CSFP and the normal control groups. When viewed from another perspective, atherosclerosis in patients with CSFP could be influenced by various cardiovascular risk factors; however, no statistically significant intergroup differences were observed in previous studies [7, 30]. Furthermore, in this study, conventional cardiovascular risk factors were also comparable between groups. Thus, these factors can be excluded as factors associated with the pathogenesis of CSFP. Risk factors for CSFP need further thorough research.

A previous study showed that ALP is a novel biomarker (an acute phase inflammatory reactant) [31] for cardiovascular events [14]. Both, serum ALP and CRP have been shown to be directly and significantly associated with each other, suggesting that they may share common biological pathways of action [32, 33]. Evidence available in this context suggests that inflammation plays an important role in the development of CSFP [8–11]. The elevated serum ALP levels observed in the CSFP group indirectly reflect an increased inflammatory reaction, which may serve as an important pathogenetic contributor to CSFP. However, it is difficult to distinguish whether this finding is a cause or a result of CSFP. Further investigations are needed to confirm these results.

Elevated serum ALP levels can cause CSFP through the action of various pathways as discussed earlier. Further studies are warranted to evaluate the role of ALP inhibitors that lower elevated serum ALP levels. Patients presenting with elevated serum ALP levels and normal angiographic findings need further investigation and close monitoring in a clinical setting.

### Study limitations

Firstly, inflammatory mediator, such as C-reactive protein, was not measured. Further more, we measured only the tissue nonspecific ALP level and did not measure the subgroups of ALP and. Last but not least, relatively few patients were included in this study. Additionally, the large sample multi-centre randomized controlled trials are needed to confirm our results.

## Conclusions

Patients with CSFP have higher serum ALP levels, and this may play an important role in the pathogenesis of CSFP. Serum ALP level was an independent predictor of the presence of CSFP. Multivessel involvement may be more severeand perhaps a worse prognosis. Patients with a higher serum ALP level may need further clinical evaluation and close monitoring. Further studies are needed to confirm these results and to investigate the treatment method from this respect.

## Abbreviations

ALP: Alkaline phosphatase
CRP: C-Reactive protein
CSFP: Coronary slow flow phenomenon
LAD: Left anterior descending
LCX: Left circumflex artery
mTFC: Mean TIMI frame count
RCA: right Coronary artery
TIMI: Thrombolysis in myocardial infarction
